# Is Immunotherapy a Contraindication for Treating Lung Cancer Patients with Interstitial Lung Diseases? A Review of the Literature

**DOI:** 10.3390/jcm15030996

**Published:** 2026-01-26

**Authors:** Raffaella Pagliaro, Paola Della Monica, Vito D’Agnano, Angela Schiattarella, Antonio D’Orologio, Paola Maria Medusa, Giulia Maria Stella, Federica Colapietra, Fabio Perrotta, Andrea Bianco, Marina Di Domenico, Filippo Scialò

**Affiliations:** 1 Department of Translational Medical Sciences, University of Campania Luigi Vanvitelli, 80131 Naples, Italy; raffaella.pagliaro@studenti.unicampania.it (R.P.); vito.dagnano@studenti.unicampania.it (V.D.); angela.schiattarella1@studenti.unicampania.it (A.S.); antonio.dorologio@studenti.unicampania.it (A.D.); paolamaria.medusa@unicampania.it (P.M.M.); fabio.perrotta@unicampania.it (F.P.); 2 Unit of Respiratory Diseases “L. Vanvitelli”, A.O. dei Colli, Monaldi Hospital, 80131 Naples, Italy; 3 Department of Precision Medicine, University of Campania Luigi Vanvitelli, 80138 Naples, Italy; paola.dellamonica@unicampania.it (P.D.M.); federica.colapietra@unicampania.it (F.C.); marina.didomenico@unicampania.it (M.D.D.); 4 Department of Internal Medicine and Medical Therapeutics, University of Pavia, 27100 Pavia, Italy; g.stella@smatteo.pv.it; 5 Respiratory Diseases Unit, Cardiothoracic and Vascular Department, IRCCS San Matteo Polyclinic Hospital, 27100 Pavia, Italy; 6 Department of Molecular Medicine and Medical Biotechnologies, University of Naples “Federico II”, 80131 Naples, Italy; filippo.scialo@unina.it

**Keywords:** lung cancer, interstitial lung disease, immune check point inhibitors, antifibrotic treatment

## Abstract

The management of lung cancer (LC) in patients with interstitial lung diseases (ILDs) presents significant challenges, particularly with the increasing use of immunotherapy (IT). Immunotherapy-related pneumonitis (ICIP) is a potential complication of immune checkpoint inhibitors (ICIs) that can be difficult to differentiate from pre-existing or treatment-induced ILD. The incidence of treatment-related pneumonitis is higher in patients with pre-existing ILD, which complicates the therapeutic approach. Moreover, antifibrotic drugs have shown potential in reducing the incidence of post-operative acute exacerbations in IPF patients undergoing surgery and radiotherapy. ILDs in LC patients can either develop ab initio, linked to environmental exposures, autoimmune diseases, or emerge because of cancer therapies. Although large-scale clinical trial evidence remains limited, careful therapy selection, early detection of pneumonitis, and close monitoring are crucial. Further prospective studies are needed to refine therapeutic strategies, particularly regarding the role of IT in this sensitive population and the role of combination therapies with antifibrotics and ICIs to optimize outcomes for patients with both LC and ILDs. This review summarizes the available evidence on the safety and efficacy of IT in this population, emphasizing the importance of personalized treatment approaches and vigilant monitoring.

## 1. Introduction

Interstitial lung diseases (ILDs) are a heterogeneous group of lung disorders characterized by inflammation and/or fibrosis of the lung parenchyma [1]. The etiology of ILD is heterogeneous with some identified risk factors such as cigarette smoking (smoking-related ILDs), environmental causes or drugs (exposure-related ILDs) [2], and autoimmune diseases. These factors can increase the likelihood of developing interstitial lung disease in genetically susceptible individuals [3].

**Figure 1:**
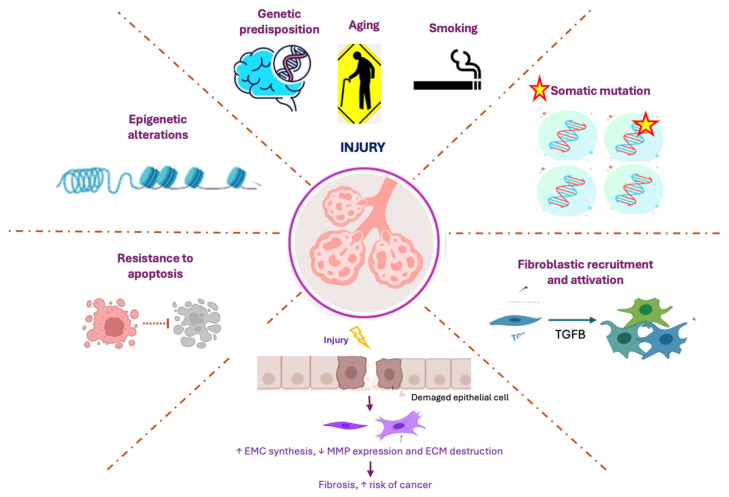
Molecular mechanisms of tissue injury and pathogenesis in LC and IPF.

Remarkably, ILD patients are also at an increased risk of developing lung cancer (LC) [4]. Several mechanisms have been postulated linking carcinogenesis in ILD patients including inflammatory mediators’ production, exhaustion of the surveillance immune cells, and activation of common profibrotic and proliferative pathways [5]. More exhaustive data are present in IPF patients who share some key contributing factors with LC, including genetic predisposition, aging and cigarette smoking, leading to persistent cellular damage [6]. This damage leads to the accumulation of somatic mutations that trigger the activation and recruitment of fibroblasts, resulting in excessive fibrosis, which is a hallmark of IPF [7]. Likewise, the epithelial–mesenchymal transition (EMT), a process that enhances fibrosis in IPF and promotes tumor invasiveness in LC, plays a critical role in both diseases by facilitating tissue remodeling and increasing cancer risk [8,9,10]. Furthermore, the resistance to apoptosis in fibrotic and transformed cells allows for their persistence, contributing to disease progression [11]. Epigenetic alterations, which regulate gene expression in response to both genetic and environmental insults, further exacerbate the pathogenesis of LC and IPF by influencing cellular behavior and promoting an aberrant response to injury [12,13,14]. These overlapping molecular mechanisms underscore the complex nature of both diseases and highlight potential therapeutic targets (Figure 1).

In patients with underlying ILDs, significant differences in LC histotype and activating mutations, when compared to general population, have been reported. Squamous cell carcinoma is the more prevalently reported carcinoma in published cohorts of IPF patients [15], while in patients with systemic autoimmune disorders, the adenocarcinoma subtype is more prevalent, in line with the general population [16].

Diagnosing LC in patients with ILD can be particularly complex, especially given the absence of established LC screening programs. Depending on the type of ILD, it can be difficult to distinguish a lung nodule from areas of fibrosis. Percutaneous lung needle biopsy also carries increased risks of complications in patients with fibrosing lung disease [17,18,19]. In this setting, artificial intelligence may provide a valuable tool to support the detection and differential diagnosis of nodules [20,21]

The development of ILDs in LC patients can occur either ab initio or during treatment, presenting distinct challenges for their management [22]. ILDs that arise ab initio are typically linked to environmental exposures, genetic predispositions, or autoimmune conditions, often diagnosed prior to the onset of cancer symptoms [23]. In contrast, ILDs that develop during treatment are often related to specific cancer therapies, such as certain chemotherapeutic agents, radiation therapy, or immunotherapy, which can trigger or exacerbate pre-existing lung conditions [24,25]. The distinction between these two pathways is critical, as the onset of treatment-related ILDs may present acutely and require immediate attention and need to be differentiated from cancer progression or treatment-related toxicity [9,26]. Understanding these differences is essential for tailoring effective treatment strategies and improving patient outcomes. To better frame the development of this review, it should be noted that, although data are limited, the distinction between IPF and non-IPF remains the most clinically relevant classification in the studies included.

Hence, we provide a comprehensive overview of the key considerations for the treatment of patients with NSCLC and ILD, with a particular focus on the risks of lung toxicity associated with ICIs and the importance of monitoring for ILD progression during cancer treatment.

The figure illustrates the key biological and molecular mechanisms involved in the pathological processes of LC and IPF. Genetic predisposition, aging, and smoking are major factors contributing to cellular damage. This damage leads to somatic mutations which trigger the recruitment and activation of fibroblasts, resulting in excessive fibrosis. The process of EMT enhances both fibrosis (in IPF) and tumor invasiveness (in LC), thereby increasing cancer risk. Moreover, resistance to apoptosis allows for abnormal and fibrotic cells to persist, while epigenetic alterations regulate gene expression in response to genetic and environmental insults, further promoting the pathogenesis of both diseases.

## 2. Lung Cancer Management in Patients with Interstitial Lung Diseases: Balancing Risks and Benefits

### 2.1. Systemic Treatment: Chemo-Immunotherapy vs. Immunotherapy Alone in ILD Patients with NSCLC

**Figure 2:**
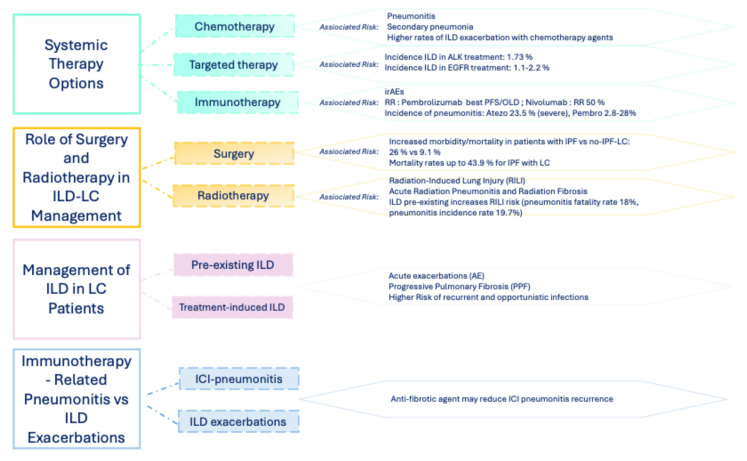
Management of lung cancer in patients with interstitial lung diseases.

The selection of appropriate systemic therapy regimens for patients with both NSCLC and ILD is quite complex (Figure 2). The recommended approach would be to select treatments that improve the quality of life of patients for the duration of survival. However, chemotherapy (CT) increases vulnerability to secondary pneumonia as immunity is suppressed and may increase morbidity and even mortality in patients with limited respiratory reserves [27]. In general, the mortality rate of CT-related pneumonitis is reported as approximately 30% and is believed to be the most common cause of treatment-related death [28]. Moreover, the incidence and mortality rates of immunotherapy-related pneumonitis (ICIP) are higher in patients with pre-existing ILD [29,30]. For systemic treatment, two Japanese phase II studies evaluated the combination of carboplatin and nab-paclitaxel in NSCLC patients with ILD, showing a low incidence (4%) of treatment-related AE-ILD [31,32]. A meta-analysis of 684 patients with ILD and advanced NSCLC undergoing first-line CT reported a 43% response rate (RR), with ILD exacerbation rates higher in treatments with docetaxel (28%) and gemcitabine (43%), while vinorelbine showed no AE-ILD in a small study [33].

Nevertheless, a retrospective study of patients with LC and IP reported an IP-AE rate of 28.6% for pemetrexed used as a second-line therapy [34]. This suggests that caution should be exercised when using pemetrexed-based regimens due to the risk of IP-related adverse events.

According to a review of the literature, the incidence of pneumonitis during IT treatment is higher in NSCLC compared to other cancers and treatment with IT alone carries a lower risk of immune-related adverse events (irAEs) compared to the combination with immune-chemotherapy. However, across different clinical settings, IT treatment is better tolerated than combination regimes, with a lower incidence and severity of ICI-adverse events [35]. In addition, the treatment for LC, pre-existing ILDs, smoking history, and male sex appear to increase the risk of ICIP [36,37].

In terms of IT therapy, when comparing prognosis by ICI agent, pembrolizumab demonstrated the best median PFS and OS compared to other ICIs. This could be related to the high levels of PD-L1 expression in patients who received pembrolizumab in this study: 63.2% (43/68) of these patients had a PD-L1 expression of ≥50% [38,39]. Yamaguchi et al. reported no significant difference in the prognosis based on the presence or absence of pre-existing IP in patients with NSCLC with PD-L1 expression ≥ 50%. Therefore, patients with a high PD-L1 expression in LC and IPF could be favorable candidates for pembrolizumab monotherapy [40].

Moreover, Yamaguchi et al. explored the atezolizumab (an anti-PD-L1) as a single arm in patients with NSCLC with pre-existing ILD and showed that 23.5% developed severe pneumonitis of grade 3 or higher, and the study was discontinued [41]. Although the results showed that nintedanib could be combined with atezolizumab while suppressing symptomatic pneumonitis, safety cannot be overemphasized based on a case series of only four patients [41].

Another pilot trial showed the promising efficacy of nivolumab in patients with NSCLC and ILD, reporting a high RR of 50% [42]. The observed efficacy in the ILD group may be attributed to the higher proportion of patients without EGFR mutations or those with a history of smoking, both of which have been associated with better treatment outcomes in patients treated with ICIs [43,44]. The additional explanation may be derived from the tumor mutation burden (TMB) and, in particular, a high TMB is associated with a better response to nivolumab [45]. Considering these data, the treatment with nivolumab might become a promising option for patients with both NSCLC and ILDs. Nevertheless, monitoring for the early detection of pneumonitis is essential. Further prospective studies are necessary to assess the efficacy and safety of nivolumab in these patients [33].

Pembrolizumab has been approved for advanced NSCLC, either alone or in combination with CT, with the incidence of pneumonitis ranging from 2.8% to 28% [37]. Overall, while pre-existing ILD is an independent risk factor for severe pneumonitis, these risks do not diminish the overall positive outcomes of pembrolizumab treatment in LC patients, including those with concomitant ILD [23,37].

However, the higher frequency of ICIP associated with durvalumab (an anti-PD-L1 agent) may be linked to its use as maintenance therapy following radiation treatment. The Pacific trial reported an overall ICIP incidence of 33.9% for all grades [46]. In contrast, the KEYNOTE 189 study reported a lower incidence of ICIPs with 4.4% for all grades and 2.7% for ≥grade 3 [47]. Despite the risks, IT remains a valuable option for patients with NSCL and ILDs, but further prospective studies are needed to better understand its safety and efficacy in this clinical setting.

Finally, treatment response may also be influenced by the patient’s nutritional status. Consistent with previous reports, malnutrition has been associated with poorer prognosis in LC patients [48]. Malnutrition may also exacerbate systemic inflammation, oxidative stress, and metabolic dysregulation, including alterations in adiponectin levels, which could in turn affect disease progression and therapeutic outcomes [49]. In patients with fibrotic ILDs, overall adiponectin levels were not associated with disease progression, although the high-molecular-weight isoform was significantly reduced [50].

### 2.2. Surgery in NSCLC Patients with ILD: Clinical Implications for Patients with ILDs

Lobectomy with mediastinal lymph node dissection remains the gold standard for early-stage NSCLC in the general population [51,52]. However, the role of surgery in patients with a concomitant diagnosis of ILD is controversial because of the substantially higher peri-operative risk [23].

A retrospective study of 711 patients with NSCLC documented increased post-operative morbidity and mortality for patients with versus without IPF (26% vs. 9.1% and 8% vs. 0.8%, respectively) [53]. Moreover, another retrospective study of 1763 patients with ILD and NSCLC treated with lobectomy or sublobar resection documented AE in 9.3% of cases and mortality up to 43.9%. The risk of post-operative AE increases with age greater than 75 years, IPF versus other types of ILD, and honeycombing on CT [54]. It is essential to reveal peri-operative strategies that can reduce the risk of AE including the delivery of lower oxygen concentrations, avoidance of intra-operative lung hyperinflation, with careful attention to barotrauma and mean airway pressures, use of corticosteroids, and prophylactic antibiotics because of the risk of pneumonia [53].

Beyond the immediate post-operative period, the prognosis of patients with IPF and NSCLC remains unfavorable. The median overall survival for patients with IPF undergoing lobar or sublobar resection of NSCLC is 42 months compared to 90 months for those without IPF [55]. A systemic review of NSCLC outcomes in patients with ILDs showed that the 3-year overall survival following surgical resection was 31 to 75% for patients with ILD compared to 79 to 95% for those without ILD [56]. A retrospective analysis of 1763 patients with stage IA NSCLC and IPF documented a 5-year overall survival of 33.2% [53].

Antifibrotic drugs also appear to affect the incidence of post-operative AE in patients with IPF and NSLC. In patients receiving pirfenidone after both wedge and anatomic resections, the incidence of AE was 3.2% compared to 21.1% in those not receiving pirfenidone [57].

### 2.3. Radiotherapy in ILD Patients

Radiotherapy represents an alternative local treatment option for patients with early-stage NSCLC [58], particularly when surgery is contraindicated; however, its use requires the careful consideration of radiation-induced lung toxicity (RILI), especially in patients with underlying ILD, which is typically caused by exposure to high-dose ionizing radiation. This side effect results in damage to the DNA of healthy tissue such as alveolar epithelial cells in addition to tumors. RILI is characterized by a marked elevation in inflammatory cytokines such as IL-4, IL-6, IL-8, and tumor necrosis factor (TNF) alpha, as well as profibrotic cytokines such as transforming growth factor (TGF) beta [59]. Moreover, RILI can be divided into two overlapping phases: the acute phase, where radiation pneumonitis is more prominent, and the late phase, which is characterized by radiation fibrosis [59]. Furthermore, it is crucial to assess the combined effects of thoracic radiation and ICIs, as this may influence the risk of developing pneumonitis. Radiation pneumonitis can sometimes be distinguished from ICIs pneumonitis based on the location of lung injury in relation to radiation isodose curves [60]. However, ILDs are known to increase the risk of pneumonitis after radiotherapy [61].

ILD substantially raises the risk of developing radiation pneumonitis, with fatal pneumonitis rates reaching up to 18%. In one study, 80% of patients who developed severe pneumonitis due to thoracic radiation showed signs of ILD on their pre-radiotherapy thoracic imaging [60]. Furthermore, thoracic radiation increases the risk for exacerbations of ILD, particularly IPF.

In an analysis of 66 patients involving treatment with SBRT of primary and metastatic lung tumors, the presence of subclinical ILD was identified as the sole factor significantly linked to the occurrence of grade 2 to 5 radiation pneumonitis [62]. A systematic review compiling these findings reported a median incidence of grade ≥ 3 radiation pneumonitis of 19.7% (range: 8–46%) in patients with ILDs receiving radiation therapy for LC. Patients treated with particle beam therapy or stereotactic ablative radiotherapy had a lower incidence (median 12.5%) than those treated with conventional radical radiotherapy (31.8%). Grade 5 radiation pneumonitis occurred with a median rate of 11.9% (range: 0–60%) [63]. The existence of ILD independently predicted severe radiation pneumonitis. According to a recently published retrospective, multicenter European study, only a small percentage of 12.5% of patients diagnosed with both IPF and LC received radiotherapy [9].

Recently, a retrospective cohort study from a UK tertiary oncology center evaluated 1693 patients referred for curative-intent thoracic radiotherapy, of whom 163 underwent specialist radiological review (53 ILD, 53 interstitial lung abnormalities (ILA), and 57 without radiological evidence of ILD/ILA). The median overall survival differed significantly across groups (9.4 months for ILD, 14.7 months for ILA, and 22.5 months for no ILD/ILA), with ILD independently associated with worse survival. Treatment-related toxicity was substantial, with grade 5 pneumonitis occurring in 13% of ILD patients and 6% of ILA patients compared with 0% in patients without ILD/ILA and conventional radiotherapy associated with higher adverse event rates than hypofractionated regimens [64]. In this contest, a recent multidisciplinary consensus addressed the management of LC patients with ILD undergoing thoracic radiotherapy, highlighting the absence of standardized guidelines and the need for careful patient selection. Using a structured, expert-based approach, the consensus emphasized the importance of comprehensive clinical, radiological, and functional assessment, multidisciplinary decision-making, accurate evaluation of toxicity risk, and individualized risk–benefit analysis when radiotherapy is delivered alone or in combination with systemic therapies [65].

### 2.4. Systemic Treatment for ILD Patients with Oncogene-Addicted NSCLC

Targeted therapies represent an important treatment option for patients with NSCLC who are not candidates for surgery and harbor actionable driver mutations, such as Epidermal Growth Factor Receptor (EGFR), Anaplastic Lymphoma Kinase (ALK), or BRAF alterations [66]. Overall, pulmonary toxicity is rare, with most using targeted agents [67]. However, even though these events are usually mild and often resolve with dose reduction or treatment discontinuation, their recognition remains essential when evaluating treatment-related complications. In a retrospective study in patients with NSCLC treated with Tyrosine Kinase Inhibitor (TKI), the incidence of pneumonitis in 1840 patients included in that study is around 1.73%. Notably, pre-existing IPF was associated with an increased risk of TKI- induced pneumonitis (6.2% vs. 0.8%, *p* = 0.0014) [68].

Targeted therapies may also lead to ILD or drug-induced pneumonitis, with reported incidence ranging from approximately 4% with gefitinib to 2.1% with ALK inhibitors [69]. For ALK TKIs, the median time to ILD onset is 53 days, with over 70% of adverse events occurring within the first two months of treatment [70].

Importantly, drug-induced pneumonitis is more frequently reported in Asian populations, particularly in Japanese cohorts, whereas severe events, including grade 5 toxicity, are significantly less common in non-Asian populations, suggesting the influence of genetic or environmental susceptibility factors [71]. Despite these risks, targeted therapies remain an option for treatment in patients with NSCLC and coexisting ILD, with special considerations for shorter follow-up to be applied. Data from a large retrospective cohort involving 555 patients with NSCLC revealed that 31 patients had pre-existing ILD, while EGFR mutations were detected in 246 patients (46%). A significant inverse correlation was observed between the presence of EGFR mutations and ILD, with only 1 out of 246 EGFR-mutant tumors arising in patients with ILD compared to 30 of 309 tumors in the EGFR wild-type group (*p* < 0.001) [72]. Moreover, no significant differences have been reported in the incidence of ILD between generations of EGFR-TKIs (gefitinib 1.3–2.2%, erlotinib 0.6–1.5%, afatinib 0.2–0.6%, and osimertinib 3.0%), with an overall frequency ranging from 1.1 to 2.2% [73,74].

From a pathophysiological perspective, TGFβ plays a key role in the development of IPF by promoting EMT; in this process, normal alveolar epithelial cells can transform into fibroblast-like cells, contributing to extracellular matrix deposition and fibrotic remodeling [75]. However, EMT has also been implicated in the development of resistance to EGFR-TKIs in NSCLC [76]. This resistance may be associated with decreased expression of E-cadherin and the upregulation of mesenchymal markers such as N-cadherin and vimentin [77]. Among the signaling pathways involved, Notch-1 activation has been shown to promote EMT and contribute to EGFR-TKIs resistance [78,79]. In this context, EMT represents a dual therapeutic challenge, contributing both to an increased resistance to targeted therapies and enhanced risk of pulmonary toxicity.

### 2.5. Management of ILDs in Lung Cancer Patients: Balancing Risks and Benefits

ILD complicates the management of LC due to difficulties in interpreting radiological findings and an increased risk of treatment-related toxicities, such as acute exacerbations and pneumonitis from various therapies. Limited evidence-based treatment options exist for patients with both ILD and LC, stemming from a lack of large, phase III randomized controlled trials. To enhance outcomes in this patient population, there is a need for emerging diagnostic modalities, biomarkers, and optimized personalized treatment strategies, with antifibrotic treatments potentially playing a role in mitigating pulmonary toxicity, though their effectiveness remains uncertain [9].

Inflammatory cells in ILD produce cytokines that stimulate epithelial cell proliferation, leading to genetic alterations such as p53 mutations, documented as the most common mutation in ILD-associated lung cancer. Aberrant pathways, including those involving tyrosine kinases and TGF-β, promote tumor growth and aggressiveness in LC patients with IPF, with nintedanib showing efficacy for both conditions. Additionally, altered methylation patterns and an elevated expression of PD-L1 contribute to immune suppression and challenge the management of LC in this patient population [53].

Patients with LC are recognized to have a higher risk of recurrent infections, including opportunistic infections, due to comorbidities and immunosuppression from cancer treatments [80]. Following acute lung damage, pro-inflammatory cytokines such as TNF-α, IL-1β, IL-8, and IL-6 are the first to be released, playing a key role in the inflammatory response [81]. The treatment with ICIs may disrupt T-cell-mediated immunity, potentially leading to excessive inflammation [82]. In general, the lung mucosal tissue in LC patients is colonized by a diverse bacterial community, which is linked to clinical outcomes. This bacterial colonization contributes to lung adenocarcinoma-related inflammation by stimulating lung resident γδ T cells [83]. In the context of ILDs, Wootton et al. found that there was no clear infection trigger for AE of IPF in patients with LC; they tested bronchoalveolar lavage (BAL) fluid and serum from patients with AE of IPF, stable IPF, and acute lung injury (ALI) for viral nucleic acids. Among 43 patients with AE, only four showed evidence of common respiratory viruses, while the remaining infections were associated with rarer pathogens [84].

## 3. Immunotherapy-Related Pneumonia vs. Progressive ILDs: A Comparison of Their Characteristics, Diagnosis, and Management in Patients with Lung Cancer

Based on the results of the epidemiological studies, a significant proportion of hospitalizations in ILD patients were related to ILD progression, exacerbation, or other reasons, causing acute worsening of respiratory symptoms [85]. In general, management of the different forms of acute presentation [86] is complex, and the incidence of AE of ILD ranges from 1.9 to 11.7% per year [85]. The European consensus group for ILD has revised the diagnostic criteria for AE-IPF with the following characteristics: a previous or concurrent diagnosis of IPF; an acute worsening or development of dyspnea, typically of <1 month duration; a CT scan with new, bilateral ground-glass opacities (GGOs) and/or consolidation superimposed on a background pattern consistent with the usual interstitial pneumonia pattern; and, at last, a deterioration not fully explained by cardiac failure or fluid overload [87]. In contrast, for differential diagnosis in these patients, it is essential to evaluate the progressive pulmonary fibrosis (PPF) defined by the worsening of clinical, functional, and radiological symptoms occurring within the past year without alternative explanation [88].

The clinical presentation of AE or PPF can be highly variable and often difficult to distinguish from other competing pulmonary diagnoses like ICIP [89].

A recent meta-analysis comparing PD-1 and PD-L1 inhibitors showed that both classes significantly increase the risk of ICIP; however, PD-1 inhibitors were associated with a higher risk than PD-L1 inhibitors, although this difference was attenuated when combined with chemotherapy [90].

The clinical presentation of ICIP can be highly variable and often difficult to distinguish from other competing pulmonary diagnoses. Common symptoms include cough, dyspnea, fatigue, and decreased activity tolerance; therefore, it can be hard to distinguish from symptoms of pre-existing ILD or its exacerbation independent of ICI therapy [60,91]. Whenever ICIP is suspected, computed tomography of the chest should be performed, since chest radiography lacks sensitivity or specificity to make the diagnosis. The most common radiological pattern of ICIP is organizing pneumonia (OP), but other patterns that can occur include non-specific interstitial pneumonitis (NSIP), hypersensitivity pneumonitis (HP), acute interstitial pneumonia (AIP), and others [92].

Nevertheless, the treatment of ICI pneumonitis is determined by its severity; asymptomatic (grade 1) pneumonitis only requires the temporary interruption of ICI therapies, while grade 4 pneumonitis requires high-dose corticosteroids and additional immunosuppressive therapies [93]. ICI-induced pneumonitis is classified as a drug-induced ILD, and its underlying pathobiology may not be the same as that of pre-existing ILD. This distinction can impact the duration of treatment and the likelihood of recurrence, even when the radiologic or histologic patterns appear similar [94].

In this context, recognizing the acute event that has contributed to the patient’s clinical worsening is essential to be able to guide the correct management. However, drug rechallenge after pneumonitis remains an area of uncertainty. According to the current guidelines, ICI rechallenge may be considered in patients with grade 2 pneumonitis, but it is mandatory to discuss every case for better management [95,96]. Evidence from the literature remains limited. In a multicenter retrospective study by Osaka et al., 32 patients who were rechallenged after ICIP experienced a high recurrence rate; however, patients with pre-existing ILD were not specifically analyzed [97]. Similarly, a small study by Wu et al. reported a recurrence rate of approximately 50% following ICI rechallenge. No significant differences in survival outcomes were observed between patients with and without recurrence. These findings highlight the need for further validation, particularly in patients with pre-existing ILD [98]. However, the current consensus recommends following patients 4–6 weeks after ICIP occurs or after hospital discharge to guide the steroid taper, followed by an additional assessment to confirm the complete resolution of clinical symptoms and radiological abnormalities [99].

Moreover, cytokine profiling through bronchoalveolar lavage (BAL) shows promise in the diagnosis and pathophysiology of ICIP with a key role played by IL-6 and Th17 cells [100].

The literature highlights the potential of combination therapies, particularly with nintedanib, due to its antifibrotic effects, which may make it a promising option when used alongside ICIs. However, there is limited evidence showing that nintedanib reduces the risk of pneumonitis in this context, with only one reported case where a patient with recurrent ICI-induced pneumonitis avoided further pneumonitis after being treated with nintedanib and atezolizumab; indeed, preclinical and early clinical studies indicate that nintedanib may exert anti-tumor and immunomodulatory effects, potentially supporting ICI efficacy in patients with LC and fibrotic ILD [101]. However, evidence in humans remains extremely limited. Interactions between antifibrotics and ICIs, including any potential protective effects against AE-ILD or ICIP, are not well understood. Moreover, a recent retrospective study [102] provides new insights into the interaction between antifibrotic therapy and efficacy outcomes in patients with fibrotic ILD who develop LC. Clinically, the continuation of antifibrotics during cancer treatment appears feasible, as no increase in AE-ILD was observed. While a definitive protective effect could not be demonstrated, the absence of safety concerns supports individualized continuation, especially in patients with progressive ILD. Antifibrotics should not be considered a contraindication to immunotherapy, but heightened monitoring for pulmonary toxicity is advised. Overall, these findings are hypothesis-generating; prospective, controlled studies are needed to clarify the safety and efficacy of combining antifibrotics with ICIs, and treatment decisions should be individualized based on patient risk and tolerance.

## 4. Conclusions

The use of IT in LC patients with pre-existing ILDs presents significant clinical challenges and requires careful consideration. While IT has improved outcomes for many cancer patients, its application in those with ILD is controversial. Most clinical trials that established the efficacy of systemic therapies for NSCLC excluded patients with ILD, leading to a limited understanding of how chemotherapy interacts with underlying ILD. However, chronic inflammation, common in both pulmonary fibrosis and cancer development, may create a microenvironment that fosters cancer progression. While some studies suggest IT may worsen ILD symptoms and lead to severe respiratory complications, others show that selected patients with stable ILD may benefit from IT without significant lung function decline. In this clinical context, it is essential to individualize treatment for each patient by leveraging prospective real-world studies integrated with validated biomarkers in order to predict and manage adverse events early, improving patient outcomes and treatment efficacy. Collaborative decision-making among oncologists, pulmonologists, and patients is crucial in balancing the benefits of immunotherapy with the risks of exacerbating ILD. In summary, ICI selection in ILD patients should integrate radiological assessment of ILD, pulmonary function evaluation, and clinical factors including comorbidities, PD-L1 expression, prior treatments, and risk factors for pneumonitis, aiming to maximize efficacy while minimizing the risk of severe CIP. Close monitoring for early signs of pneumonitis is essential regardless of the agent chosen.

## Data Availability

The original contributions presented in this study are included in the article. Further inquiries can be directed to the corresponding author.
